# Pgrmc1: new roles in the microglial mediation of progesterone-antagonism of estradiol-dependent neurite sprouting and in microglial activation

**DOI:** 10.3389/fnins.2013.00157

**Published:** 2013-09-03

**Authors:** N. Bali, T. E. Morgan, C. E. Finch

**Affiliations:** ^1^Davis School of Gerontology, University of Southern California Los AngelesLos Angeles, CA, USA; ^2^Dornsife College of Letters, Arts and Sciences, University of Southern California Los AngelesLos Angeles, CA, USA

**Keywords:** Prgmc1, microglia, progesterone, sprouting, S2R

## Abstract

Pgrmc1 (progesterone receptor membrane component 1) is a multifunctional 22 kDa protein with heme-binding and P450-activating capacity which was recognized under different names for roles in cell motility during neural development and in cancer, and apoptosis. Pgrmc1 expression in microglia was recently shown by the present authors to mediate estrogen-progesterone interactions during axonal sprouting and to mediate microglial activation itself. We also discuss other functions of Pgramc1 in the nervous system and its possible relationship to the 18 kDa sigma-2 receptor (S2R).

## Introduction

Progesterone receptor membrane component 1 (Pgrmc1) is recognized for roles in many organs from independent discoveries; in neuroscience, its names include 25-Dx and VEMA (Cahill, 2007; Kimura et al., 2012). Other family members include Pgrmc2, neudesin, and neuferricin, which are distinct from the classical progesterone receptor (PR) transcription factors and from the large family of progestin/adipoQ membrane receptors that include mPRα, mPRβ, and mPRγ. We recently discovered two new roles of Pgrmc1 in microglial activation and in the microglial mediation of ovarian steroid effects on neurite sprouting (Bali et al., 2013).

Interactions of estradiol (E2) and progesterone (P4) are fundamental to reproductive cycles. In the uterus during the ovarian follicular phase, endometrial tissue growth is stimulated by plasma E2 elevations, while in the ovary, P4 regulates apoptosis of follicular granulosa cells via Pgrmc1 (Peluso et al., 2010; Peluso, 2013). During the ovarian luteal phase, if fertilized ova did not implant, plasma P4 levels fall, causing regression of endometrial tissues. A parallel process occurs in synapses of some neuronal systems. In the rat hippocampus, E2-driven dendritic spine numbers on CA1 pyramidal neurons increase during the follicular phase, followed by spine loss during the luteal phase P4 surge (Woolley and McEwen, 1992). Unlike the uterus and ovary, hippocampal synaptic cycles do not include neuron cell death.

E2-P4 interactions also modulate axonal regeneration during compensatory neuronal sprouting of the perforant path projections from the entorhinal cortex to the dentate gyrus molecular layer of the hippocampus. In ovariectomized (OVX) rats given entorhinal cortex lesions (ECL) to axotomize the perforant path, we found that P4 antagonized E2-dependent axonal outgrowth into the dentate gyrus molecular layer (Wong et al., 2009). Glial activation in the DG molecular layer peaks at days 3–4 post-ECL. By immunocytochemistry E2 implants decreased astrocyte (glial fibrillary acidic protein, GFAP) and microglial (isolectin B4) activation, whereas P4 antagonized E2-mediated decrease in glial activation, similar to its effects on neurite sprouting. This model may be used to optimize hormone therapy for axonal maintenance during the perforant path degeneration of early Alzheimer disease (Braak et al., 2006) and for neuroprotective effects of P4 in traumatic brain injury (TBI) (Stein, 2011).

These complex neuron-glial interactions were analyzed further with the “wounding-in-a-dish” model, in which embryonic rat E18 cortical neurons are grown on confluent glia (Wong et al., 2009). Axotomy by scratch-wounding induces neurite outgrowth, which was enhanced by E2 and antagonized by E2+P4 as *in vivo* with ECL. To our surprise, the E2-P4 antagonism of neurite outgrowth occurred with mixed glia (astrocytes:microglia, 3:1), but not with enriched astrocytes (>95%). This finding was unexpected because microglia reportedly lacked expression of Pgr, the classical PR (Sierra et al., 2008). Yet, P4 antagonism of E2-dependent sprouting was blocked in mixed glia by two classical PR antagonists (ORG-31710 and RU-486) (Wong et al., 2009). Analysis of PRs of both astrocytes and microglia revealed new roles of Pgrmc1.

## Progesterone receptors in brain cells

**Figure 1:**
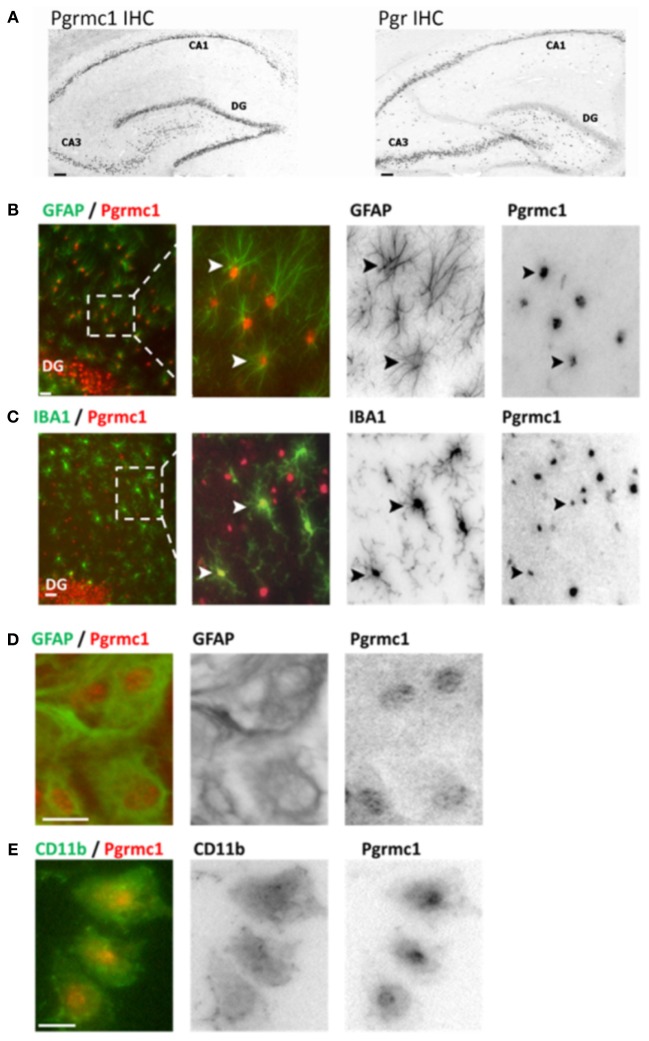
**Pgrmc1 and Pgr (PR) expression in rat brain glia and neurons by immunocytochemistry. (A)** Neuronal Pgrmc1 and Pgr expression in adult rat hippocampus was equivalent in the hippocampus CA1, CA3, and dentate gyrus (DG) layers. In contrast, neuronal Pgr expression was lower DG vs. CA1 and CA3 layers. **(B)** Astrocyte Pgrmc1 in DG molecular layer co-stained with GFAP. Arrowheads point to co-labeled astrocytes. **(C)** Microglial Pgrmc1 in DG molecular layer co-stained with IBA1. Arrowheads point to co-labeled microglia. **(D)** Astrocyte Pgrmc1 in primary cultures from cerebral cortex co-stained with GFAP. **(E)** Microglial Pgrmc1 in primary cultures co-stained with CD11b. Scale bars, 20 μm. Modified from Bali et al. (2012) and Bali et al. (2013).

Brain P4 actions are mediated by at least eight different proteins with differential expression in neurons and glia (Brinton et al., 2008). The two classic nuclear gene transcription factors PR-A and PR-B are alternate transcripts of *Pgr*, are widely expressed in neurons throughout the adult rat brain including cerebral cortex, hippocampus, and hypothalamus (Intlekofer and Petersen, 2011; Bali et al., 2012). We detected PRs in astrocytes, but not in microglia of adult rat hippocampus (*in situ* hybridization and immunocytochemistry), and in primary glial cultures from neonatal cortex by rtPCR and Western blots (Figure 1) (Bali et al., 2013). These findings confirm observations of the absence of Pgr in *ex vivo* fluorescence-sorted (FACS) adult mouse brain microglia (Sierra et al., 2008). Pgr expression in astrocytes is also shown from antagonism of the P4:E2 interactions in neurite sprouting by ORG-31710 and RU-486, classical PR antagonists (Wong et al., 2009).

Pgrmc1 was also found in microglia, astrocytes, and neurons, both *in vivo* and *in vitro* by immunocytochemistry, rtPCR, and Western blots (Figure 1). Unlike Pgr, Pgrmc1 is uniformly expressed by all hippocampal neurons (Bali et al., 2012). Although initially characterized as a membrane receptor, Pgrmc1 can reside in the cell nucleus (Peluso et al., 2010; Bali et al., 2013). We also detected other membrane-associated PRs, mPRα, mPRβ, and mPRγ, in astrocytes and microglia (rtPCR, DNA sequence confirmed; unpublished data). Meffre et al. (2013) also reported mPRα in astrocytes and microglia, but only after TBI. In spinal cord, mPRα was found in glia, but not mPRβ (Labombarda et al., 2010). We conclude that P4 actions in microglia are mediated by membrane PRs because they lack expression of the classical PRs. A specific role of Pgrmc1 in microglial activation is described below.

The neuronal side of Pgrmc1 began in 1999, under the name of VEMA, a protein that proved to regulate neuron outgrowth in mouse and nematode (Runko et al., 1999; Cahill, 2007). Its first brain steroidal association was “25-Dx,” from a cDNA encoding a 25-kDa protein that responded to E2 and P4 (Krebs et al., 2000). In hypothalamic neurons, 25-Dx induction by E2 was blocked by P4. However, in hippocampal neurons of OVX rats, P4 did not antagonize E2-induced Pgrmc1 expression; moreover, P4 was as strong an inducer of Pgrmc1 as E2 alone (Bali et al., 2012). Its hypothesized role in reproductive functions was shown in GnRH neurons, where the inhibition of [Ca^+2^]_i_ oscillations by P4 was blocked by AG-205, a Pgrmc1 ligand inhibitor, but not by RU486, the classic PR antagonist (Bashour and Wray, 2012). In neural progenitor cells, P4 was pro-proliferative via Pgrmc1 (Liu et al., 2009). On the glial side, Pgrmc1 was detected in both astrocytes and microglia, noted above. TBI also induced Pgrmc1 in adjacent astrocytes and neurons (Meffre et al., 2005).

### Pgrmc1 and the role of microglial activation in P4-E2 antagonism

As noted above, P4 antagonized E2-induced neurite sprouting in mixed glia containing 3:1 astrocytes:microglia, but the antagonism was absent from enriched astrocytes from which microglia had been removed (>95% astrocytes) (Wong et al., 2009). A caveat is that the 4 h mechanical shaking process to remove microglia could alter glial responses, e.g., hydrodynamic forces induce astrocyte aromatase (Gatson et al., 2011). The role of microglia was shown in a new protocol to add-back microglia, which restored the P4-E2 antagonism of neurite outgrowth (Bali et al., 2013).

The add-back protocol also allowed us to separately manipulate PRs in astrocytes and microglia before the mixed glia reconstitution. An astrocyte role was anticipated by the Pgrmc1 dependence of BDNF secretion (Su et al., 2012). Using siRNA, we showed that Pgrmc1 knockdown in microglia, but not in astrocytes abolished the P4-E2 antagonism of neurite outgrowth. We then found that conditioned media from scratch-wounded cultures of microglia alone sufficed to restore the P4-E2 antagonism. To identify the simplest conditions that restored P4-E2 antagonism, we used charcoal-stripped FBS containing minimal steroids. However, the soluble activity was absent from unwounded microglia, implying a role of microglial activation. The requirement for microglial activation also explained why P4 did not antagonize E2-dependent outgrowth in non-wounded cultures. A candidate for the soluble factor was TNFα, which is released by microglia and which can inhibit neurite outgrowth; however TNFα levels did not differ with scratch wounding in our model (unpublished).

**Figure 2:**
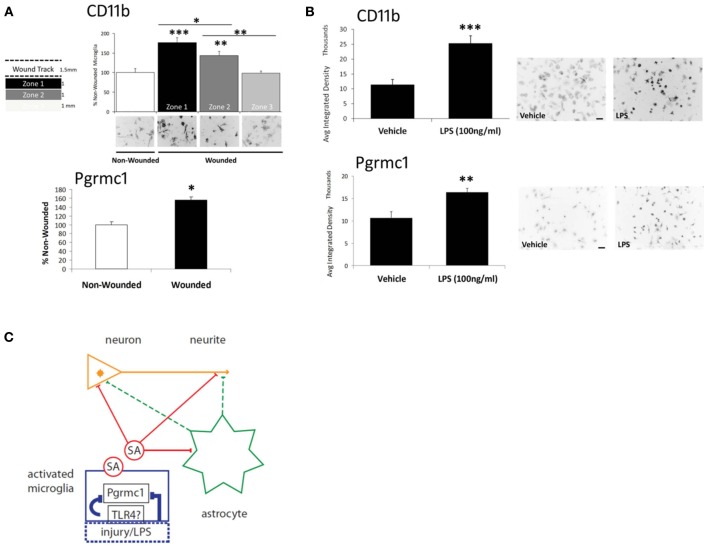
**Microglial activation via scratch wounding and LPS. (A)** Microglial CD11b in 3 zones (1 mm wide) progressively decreased distal to the wound. Pgrmc1 protein was also induced by scratch wounding. ^***^ *p* < 0.0001; ^**^ *p* < 0.03; ^*^ *p* < 0.05 vs non-wounded. **(B)** CD11b and Pgrmc1 protein were both induced by by LPS. ^***^ *p* < 0.0001; ^**^ *p* < 0.001 vs vehicle. **(C)** Model to show targets of the undefined soluble activity (SA) from activated microglia and microglial Pgrmc1 that mediate the antagonism of P4 on E2-dependent neurite outgrowth. Microglial activation by LPS is assumed to be mediated by toll-like receptor-4 (TLR4); activation by scratch wounding has undefined pathways. The schema shows two possible SA actions on neurites: direct effects (solid red lines) on neurite outgrowth at the neurite growth cone (arrowhead) and/or involving the neuronal nucleus; indirect effects (dashed green lines) via astrocyte secretions on neurite or neuronal nucleus. Glial activation by injury or LPS and the SA effects were blocked by Pgrmc1 knockdown. Modified from Bali et al. (2013).

Because scratch-wounding is an unconventional mode of microglial activation (we did not find other reports), we examined the classical microglial activator, LPS, which restored the soluble activity. Pgrmc1 protein was also induced in microglia by scratch-wounding or by LPS (Figures 2A,B), consistent with its induction in astrocytes by TBI, noted above. Moreover, Pgrmc1 knockdown by siRNA abolished LPS microglial activation by the standard CD11b marker. The expression of Pgrmc1 in DG microglia (Figure 1E) could thus mediate *in vivo* effects of P4 on neurite outgrowth.

Thus, Pgrmc1 is not only a new marker of microglial activation, but has a fundamental role in microglial activation itself. Therefore, Pgrmc1 may regulate microglial-mediated neurotoxic, and perhaps, neuroprotective activities, as well as synaptic pruning (Aguzzi et al., 2013). The responses to LPS imply a link to toll-like receptor pathways, e.g., TLR4, which mediates microglial activation by LPS (Chen et al., 2012) (Figure 2C). Its role in the activation of other monocytes is unknown. There may also be interactions of P4 and its metabolites with other receptors throughout the body, e.g., GABA receptors.

## Possible relationship of Pgrmc1 to the sigma-2 receptor (S2R)

The S2R was reportedly identified as Pgrmc1 and 25-Dx by mass spectrometry on the basis of 29 shared residues (Xu et al., 2011). Both are also heme binding proteins with high affinity for P4 (Cahill, 2007; Johannessen et al., 2011; Peluso, 2013). The S2R is of increasing interest in various research areas which do not consistently cross-reference Pgrmc1, including apoptosis, cancer metastasis, cocaine addiction, and ion channels. Of relevance to our findings, S2R mediates microglial migration up gradients of ATP *in vitro*, with pharmacological specificities that appear distinct from P4-like ligands (Cuevas et al., 2011). However, its 18 kDa size is distinctly smaller than 25 kDa Pgrmc1 (Ruoho et al., 2013), and could be an alternatively spliced form.

Whatever the case, S2R and Pgrmc1 both mediate cell migration in diverse contexts. Recall that Pgrmc1/VEMA influenced axonal outgrowth in mice and nematodes at developmental stages (Runko et al., 1999) which are not known for P4-dependence. The activation of microglia and induction of Pgrmc1 *in vitro* in the absence of exogenous P4 (see above) may be another P4-independent effect (Mir et al., 2012). However, local P4 could have a role via the cytochrome b5-like heme/steroid-binding function of Pgrmc1 which interacts with diverse binding partners, including P450 enzymes (Cahill, 2007; Ahmed et al., 2010; Kimura et al., 2012).

## Conclusions and open questions

The diverse functions of Pgrmc1 continue to expand in many fields that have little cross-talk. Further progress may depend as much on biochemistry as further elegant molecular genetics. Pgrmc1 knockout mice may become available. The connections with innate immunity through the toll-like receptors are an attractive target. The identification of proteins or other molecules in the soluble activity from conditioned media could be done efficiently. The requirement for microglial activation in the P4-E2 antagonism of neurite outgrowth further suggests that microglial activation, such as arises during aging and Alzheimer disease, might alter the absence of P4-E2 antagonism in regulation of hippocampal neuron Pgrmc1 *in vivo* (Bali et al., 2012). Given the role of Pgrmc1 in these experiments and the finding that Pgrmc1 expression influences astrocyte BDNF (Su et al., 2012), we anticipate further roles of Pgrmc1 in the complex biology of glia-neuron interactions.

### Conflict of interest statement

The authors declare that the research was conducted in the absence of any commercial or financial relationships that could be construed as a potential conflict of interest.
